# The value of neurocognitive testing for acute outcomes after mild traumatic brain injury

**DOI:** 10.1186/s40779-016-0091-4

**Published:** 2016-07-22

**Authors:** Latha Ganti, Yasamin Daneshvar, Sarah Ayala, Aakash N. Bodhit, Keith R. Peters

**Affiliations:** University of Central Florida College of Medicine, Orlando, Florida 32827 USA; New York University, New York, NY USA; St Louis University, St Louis, MO USA; University of Florida, Gainesville, FL USA; University of California, San Diego, California USA

**Keywords:** Neurocognitive testing, Mild traumatic brain injury, Treatment outcome

## Abstract

**Background:**

Traditionally, neurocognitive testing is performed weeks to months after head injury and is mostly performed on patients who continue to have symptoms or difficulties. In this study, we sought to determine whether these tests, when administered acutely, could assist in predicting short-term outcomes after acute traumatic brain injury (TBI).

**Methods:**

This is an IRB-approved prospective study of adult patients who came to the emergency department of our Level-1 trauma center with TBI. Patients were enrolled prospectively after providing written informed consent and underwent three separate neurocognitive tests: the Galveston Orientation Amnesia Test (GOAT) the Rivermead Post-Concussion Survey Questionnaire (RPCSQ) and the Mini Mental Status Examination (MMSE).

**Results:**

A lower GOAT score was significantly associated with hospitalization (*P* = 0.0212) and the development of post-concussion syndrome (*P* = 0.0081) at late follow-up. A higher RPCSQ score was significantly associated with hospital admission (*P* = 0.0098), re-admission within 30 days of discharge (*P* = 0.0431) and evidence of post-concussion syndrome (PCS) at early follow-up (*P* = 0.0004). A higher MMSE score was significantly associated with not being admitted to the hospital (*P* = 0.0002) and not returning to the emergency department (ED) within 72 hours of discharge (*P* = 0.0078). Lower MMSE was also significantly associated with bleeding or a fracture on the brain CT (*P* = 0.0431).

**Conclusions:**

While neurocognitive testing is not commonly performed in the ED in the setting of acute head injury, it is both feasible and appears to have value in predicting hospital admission and PCS. These data are especially important in terms of helping patients understand what to expect, thus, aiding in their recovery.

**Electronic supplementary material:**

The online version of this article (doi:10.1186/s40779-016-0091-4) contains supplementary material, which is available to authorized users.

## Background

In the United States, 1.7 to 3.8 million cases of traumatic brain injury (TBI) occur each year [1]. Because of the highly variable mechanisms of TBI, it is a common discharge diagnosis in the emergency department (ED) [2]. Despite the high incidence of TBI, acute outcomes following TBI are difficult to predict due to the differences in individual responses to trauma and other contributing factors that are unique to individuals. Coupled with the lack of evidential correlation between TBI and acute outcomes, determining acute outcomes requires an approach that is as unique to the individual as it is to the injury itself. To overcome some of the unknown factors associated with TBI recovery, this study examined how early neurocognitive testing can be used to determine connections between injury and outcome.

Traditionally, neurocognitive tests are administered by neuropsychologists for patients who continue to have symptoms or difficulties weeks to months after head injury. Ideally, the results of a neurocognitive test performed prior to injury would be available for comparison with the post-injury neurocognitive test results. However, considering the nature of emergency medicine, control test results are unlikely to be available. The neurocognitive tests administered in this study provide a “new baseline” that can be used to determine whether neurocognitive testing has merit as an acute predictive indicator of acute effects and outcomes following TBI.

## Methods

This study examined a subset of patients from a prospective cohort study that spanned a 10-month period from August 2012 to May 2013 [3, 4]. The study was conducted at a level one trauma center in the southeastern United States. The patients were screened in the ED. Subjects were considered eligible if they were 18 years of age or older and had sustained a head injury of any kind within 24 hours of presentation to the emergency department. Pregnant women, children, and prisoners were excluded.

Patients were enrolled prospectively after providing written informed consent and underwent 3 separate neurocognitive tests: the Galveston Orientation Amnesia Test (GOAT) [5], the Rivermead Post-Concussion Survey Questionnaire (RPCSQ) [6, 7], and the Mini Mental Status Examination (MMSE) [8, 9]. The GOAT is a 20-question instrument (Additional file 1) that is scored from 0 to 100. The RPCSQ is a 16-question instrument that examines post-concussion symptoms rated by the patient according to the increase in their frequency compared with premorbid levels. The total score is calculated based on 2 domains (cognitive and emotional-somatic) and ranges from 0 to 72. The questionnaire asks the sufferer to assess the following symptoms: headaches, feeling of dizziness, nausea and/or vomiting, hyperacusis, sleep disturbance, fatigue, tiring more easily, irritability, being easily angered, feeling depressed or tearful, feeling frustrated or impatient, forgetfulness, poor memory, poor concentration, taking longer to think, blurred vision, light sensitivity, double vision, and restlessness (Additional file 2).

The MMSE contains six domains of cognition: orientation, registration of new information, attention and calculation, recall, language and visuospatial construction (Additional file 3). The MMSE score ranges from 0 to 30. Independent variables included raw scores on each of these tests, and dependent variables included hospital admission, development of post-concussion syndrome, and 30-day readmission.

Demographic information was collected directly from the patient prior to discharge from the emergency department using standardized instruments. Head injury severity was classified using the Glasgow Coma Scale, with a GCS score of 13-15 indicating mild head injury. The post-injury symptoms that were collected included loss of consciousness (LOC), the duration of the LOC, alteration of consciousness (AOC), posttraumatic amnesia (PTA), seizure, vomiting, and headache. An AOC was considered to have occurred if the patient reported feeling dazed or confused or having difficulty thinking or if the neurologic exam revealed a decreased mental status. Data regarding the mechanism of the injury, including a fall, traffic accident, sports injury, and assault, were also collected. Two phone follow-ups were conducted after discharge, one at 3-15 days (termed early follow up) and one at 30-45 days (termed late follow up; Additional file 4). Patients were asked whether they had any symptoms suggestive of post-concussion syndrome, including headache, vomiting, dizziness, tinnitus, sensitivity to light/noise, numbness/tingling, blurred vision/diplopia/flashing lights, drowsiness, fatigue/lethargy, sadness/depression, nervousness/irritability, difficulty concentrating or remembering, sleeping problems, and feeling “slowed down,” “in a fog” or “dazed.” A positive response to any of these questions was considered to indicate the presence of post-concussion syndrome (PCS). Outcome variables included CT abnormality, hospital admission, return visit to the ED within 72 hours of discharge [10], readmission to the hospital within 30 days of discharge, and presence of PCS at early or late follow-up.

Data were entered into our Clinical and Translation Science Institute’s REDCap (Research Electronic Data Capture) database. REDCap is a secure, web-based application designed to support traditional case report form data capture. Statistical analyses were performed using JMP 10 for Mac. Normally distributed variables are presented as the means and standard deviations, while skewed variables are reported as medians and interquartile ranges (IQR).

## Results

The study cohort included 118 patients, and 55 % were male. The median age of the patients was 30.5 years (IQR 21-50 years, range 18-95 years). The racial composition of the cohort was 72 % white, 19 % black, 6 % Hispanic, 2 % Asian, and 2 % other, which is consistent with our county’s demographic make-up.

The following mechanisms of injury were reported when the patients presented to the ED: fall, 43 %; motor vehicle crash (MVC), 52 %; assault or head injury caused by being struck on the head with an object, 3 %; and head injury caused by a sports activity, 2 %.

In terms of alcohol consumption, 66 % of patients reported that they drank alcohol in general. Of these, 13 % consumed alcohol within 6 hours of the injury, 18 % within 6-18 hours of the injury, and 69 % more than 24 hours prior to the head injury. At the time of emergency department evaluation, 13 % of patients were intoxicated with alcohol, and overall 7 % had a urine dipstick positive for amphetamines (3 %), cocaine (3 %),cannabis (3 %), and opiates (6 %).

Forty-five percent of patients experienced LOC, and 60 % of patients reported alteration of consciousness. Thirty-six percent of patients experienced post-traumatic amnesia, and it was followed by anterograde PTA in 91 % of these patients and retrograde PTA in 30 % of these patients. Eight percent of patients reported vomiting, and 59 % reported a headache associated with their head injury.

Seventy-nine percent (*n* = 91) of patients underwent brain computed tomography (CT), and an abnormality was found in 30 % (*n* = 28) of these patients. The CT abnormalities included soft tissue swelling (14 %), skull fracture (2 %), and bleeding (5 %). No patients underwent surgical intervention for their head injury.

**Fig. 1:**
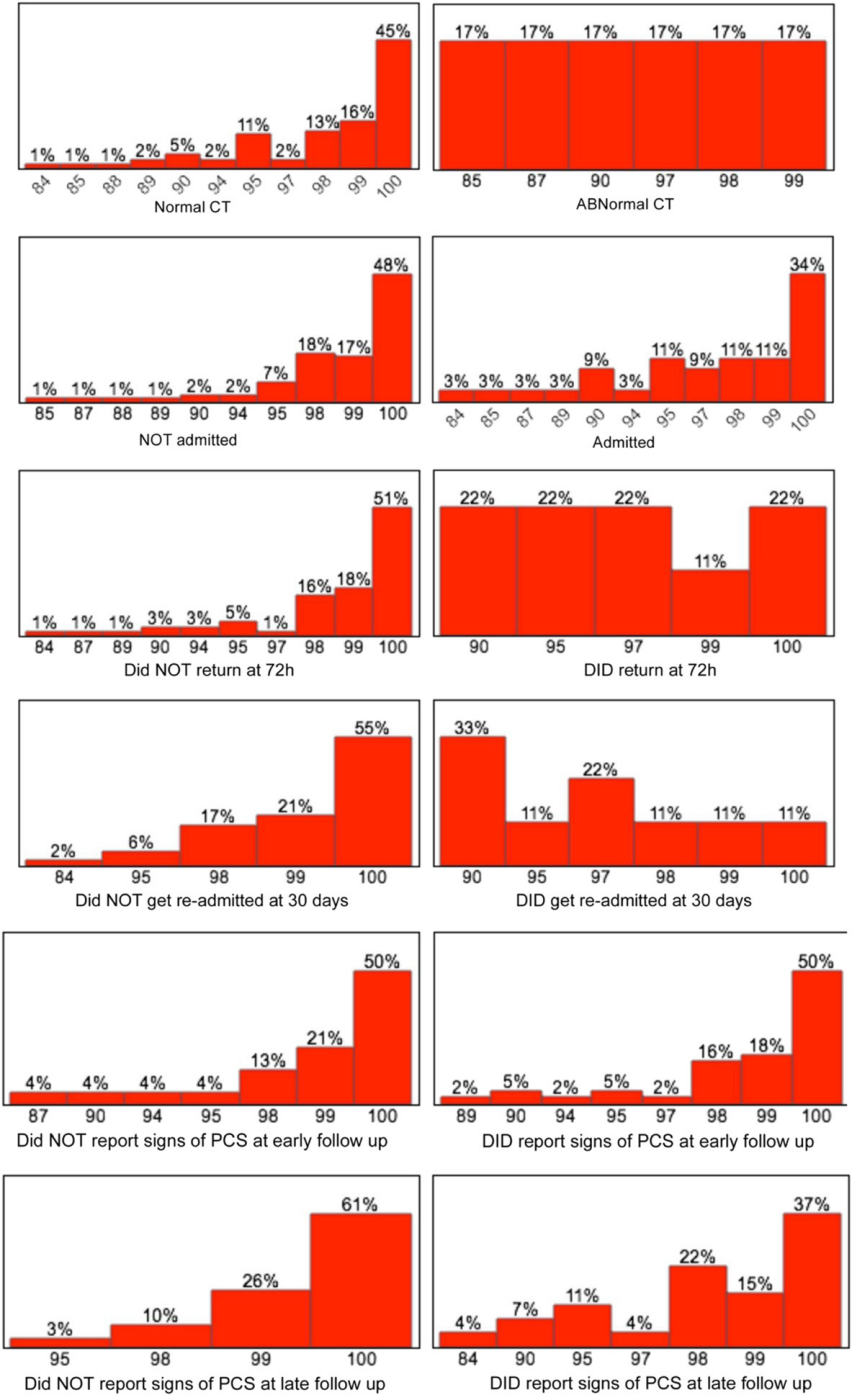
Frequency of outcomes by GOAT score

The median GOAT score was 99, with an IQR of 97.5-100 and a range of 84-100. A lower GOAT score was significantly associated with hospitalization (*P* = 0.0212) and evidence of post-concussion syndrome at the early follow-up (*P* = 0.0081, R^2^ = 11.9 %). Figure 1 summarizes the frequency of each outcome by GOAT score.

**Fig. 2:**
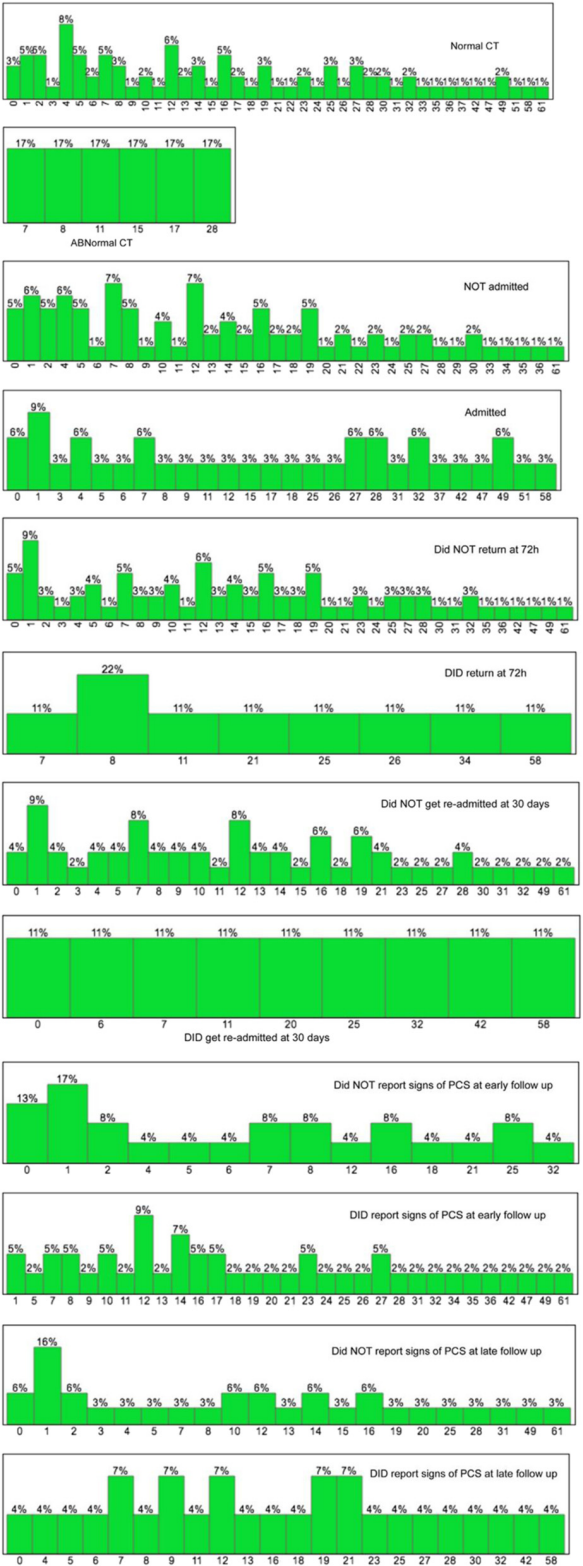
Frequency of outcomes by RPCSQ score

The median RPCSQ score was 12, with an IQR of 5-24.5 and a range of 0-61. A higher RPCSQ score was significantly associated with hospital admission (*P* = 0.0098), re-admission to the hospital within 30 days of discharge (*P* = 0.0431) and evidence of post-concussion syndrome at early follow-up (*P* = 0.0004, R^2^ = 17.2 %). In addition, a higher RPCSQ score was significantly associated with a report of LOC (*P* = 0.0470). Figure 2 summarizes the frequency of each outcome by RPCSQ score.

**Fig. 3:**
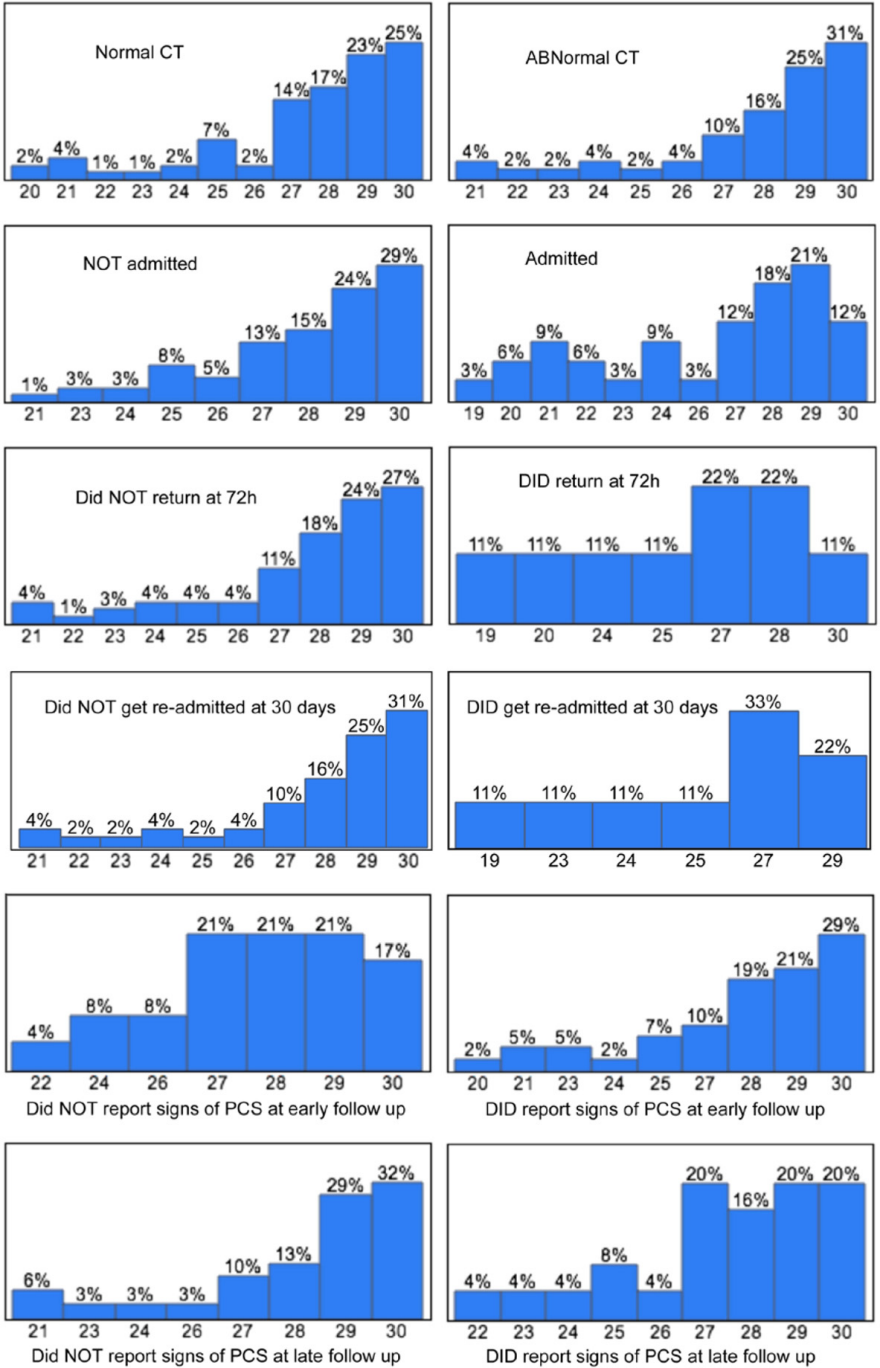
Frequency of outcomes by MMSE score

**Fig. 4:**
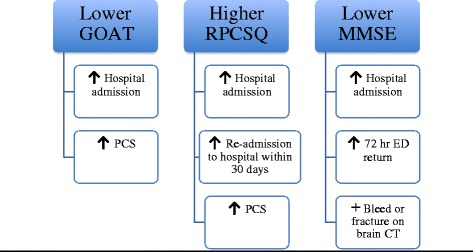
Associations of the various neurocognitive tests with patient outcomes

The median MMSE score was 28, with an IQR of 26-29 and a range of 19-30. Having a higher MMSE score was significantly associated with not being admitted to the hospital (*P* = 0.0002) and not returning to the ED within 72 hours of discharge (*P* = 0.0078). In addition, younger patients were more likely to present with a lower MMSE score (*P* = 0.0356). A lower MMSE score was also significantly associated with bleeding or a fracture on the brain CT (*P* = 0.0431). Figure 3 summarizes the frequency of each outcome by MMSE score. Figure 4 summarizes the associations of the three neurocognitive tests with patient signs and outcomes.

## Discussion

Neurocognitive testing for mild TBI in the emergency department setting is a relatively novel concept. Subjects in this cohort underwent neurocognitive testing during the course of their emergency department stay, which was within minutes to hours of their head injury. Neurocognitive tests are traditionally performed weeks to months after head injury, are usually administered by neuropsychologists and are mostly administered to patients who continue to have symptoms or difficulties after acute treatment.

The current belief is that testing may be unreliable during the hyperacute phase because the patient’s mTBI is too “fresh,” and too many other evaluations are being performed for the patient to be able to undergo neurocognitive testing. However, a more probable explanation for the lack of performance of neurocognitive testing in the ED for patients with TBI is that the evaluation of mTBI in the emergency department is relatively new, with most EDs performing little more than a brain CT, if even that is performed. The concept that emergency physicians rather than neuropsychologists can perform neurocognitive testing and that it can be performed in a busy emergency department is relatively novel.

To date, only a handful of studies have investigated the utility of neurocognitive testing in the hyperacute phase of mild traumatic brain injury. In a study of adults presenting to the ED with mTBI, the Standardized Assessment of Concussion (SAC) was administered to 66 subjects whose CT was positive for an intracranial injury [11]. The SAC is a sports sideline evaluation tool designed to determine whether a concussion has occurred and is composed of brief subtests of orientation, immediate recall, concentration, and delayed recall. The study found that the SAC score did not correlate with a positive CT scan. A study of children 10-17 years of age with and without mTBI showed that neurocognitive function, as tested in the ED using the Children Orientation and Amnesia Test (COAT), was more than 2 standard deviations lower in the group with mTBI compared to the controls, suggesting significant amnesia in the patients with mTBI [12]. A prospective cohort study of patients 11 to 17 years of age presenting to the ED within 12 hours of a head injury found that while there was no correlation for traditional concussion grading, for the neurocognitive domains of verbal memory, processing speed, and reaction time, there was a significant correlation between ED and follow-up scores trending toward clinical improvement [13]. The authors concluded that immediate neurological assessment in the ED can predict neurocognitive deficits at follow-up and has potential for individualizing management and testing different therapeutic interventions. A similar study in adults had the same findings: compared with non–head-injured patients, ED mild traumatic brain injury patients demonstrated subtle but discernible neurocognitive deficits. [14]. The current study builds on these findings by including additional neurocognitive tests in the ED and examining the following outcomes in addition to CT findings and the development of post-concussion syndrome: hospital admission, 72 hour return to the ED, and hospital re-admission within 30 days of discharge.

## Strengths and limitations

The strengths of the current study include its prospective design, the level of detail of the information obtained for each patient, the capturing of injury characteristics within the hyperacute period of the injury, i.e., within 24 hours for the entire cohort and within 12 hours for the majority of patients. The limitation of this study is that it was conducted at a single institution; thus, the patient population may have had unique characteristics. Therefore, the findings of this study may not be externally generalizable to populations that differ substantially from that of this study. However, the purpose of this paper was to demonstrate that neurocognitive testing is feasible in the ED and yields some potentially useful clinical information; therefore, the specific demographics of age and race may not be pivotal.

## Conclusions

While not commonplace, neurocognitive testing in the ED in the setting of acute head injury is both feasible and appears to have value in predicting who will suffer from PCS, hospital admission, 72 hour return to the ED, and hospital re-admission within 30 days. These data are especially important in terms of helping patients understand what to expect, which in turn can aid in their recovery.

## Abbreviations

AOC, Alteration of consciousness; COAT, Children Orientation and Amnesia Test; CT, Computed tomography; ED, Emergency department; GOAT, Galveston Orientation Amnesia Test; IQR, Interquartile ranges; LOC, Loss of consciousness; MMSE, Mini Mental Status Exam; MVC, Motor vehicle crash; PCS, Post-concussion syndrome; PTA, Posttraumatic amnesia; REDCap, Research Electronic Data Capture; RPCSQ, Rivermead Post-Concussion Survey Questionnaire; SAC, Standardized Assessment of Concussion; TBI, Traumatic brain injury

## Additional files

Additional file 1: The Galveston Orientation and Amnesia Test (GOAT). (DOC 40 kb) Additional file 2: The Rivermead Post-concussion Symptom Questionnaire (RPSQ). (DOC 1078 kb) Additional file 3: The Mini Mental Status Examination (MMSE). (DOC 246 kb) Additional file 4: Phone script for telephone follow-up. (TIFF 1798 kb)
