# Supplementary material for: The value of neurocognitive testing for acute outcomes after mild traumatic brain injury
Source: Mil Med Res. 2016 Jul 22;3:23. doi: 10.1186/s40779-016-0091-4 (PMC4957408; doi:10.1186/s40779-016-0091-4)
Supplement: Additional file 1: — The Galveston Orientation and Amnesia Test (GOAT). (DOC 40 kb) [file 40779_2016_91_MOESM1_ESM.doc]

**Appendix 1.** The Galveston Orientation and Amnesia Test (GOAT) developed by Harvey S.

| **Question** | **Error Score** | **Notes** |
| --- | --- | --- |
| What is your name? | -2 _____ | Must give both first name and surname. |
| When were you born? | -4 _____ | Must give day, month, and year. |
| Where do you live? | -4 _____ | Town is sufficient. |
| Where are you now: |  |  |
| (a) City | -5 _____ | Must give actual town. |
| (b) Building | -5 _____ | Usually in hospital or rehab center.  Actual name necessary. |
| When were you admitted to this hospital? | -5 _____ | Date. |
| How did you get here? | -5 _____ | Mode of transport. |
| What is the first event you can remember after the injury? | -5 _____ | Any plausible event is sufficient (record answer) |
| Can you give some detail? | -5 _____ | Must give relevant detail. |
| Can you describe the last event you can recall before the accident? | -5 _____ | Any plausible event is sufficient (record answer) |
| What time is it now? | -5 _____ | -1 for each half-hour error. |
| What day of the week is it? | -3 _____ | -1 for each day error. |
| What day of the month is it? (i.e., the date) | -5 _____ | -1 for each day error. |
| What is the month? | -15 ____ | -5 for each month error. |
| What is the year? | -30 ____ | -10 for each year error. |
| Total Error: |  |  |
| Total Actual Score = (100 - total error) = 100 - _____ =Can be a negative number. | | |
| 76-100 = Normal / 66-75 = Borderline / <66 = Impaired | | |

Levin, Ph.D., Vincent M. O'Donnell, M.A., & Robert G. Grossman, M.D can be administered daily.  A score of 78 or more on three consecutive occasions is considered to indicate that post-traumatic amnesia (PTA) has resolved. The instrument is freely available here: http://www.utmb.edu/psychology/Adultrehab/GOAT.htm.
