# Supplementary material for: The value of neurocognitive testing for acute outcomes after mild traumatic brain injury
Source: Mil Med Res. 2016 Jul 22;3:23. doi: 10.1186/s40779-016-0091-4 (PMC4957408; doi:10.1186/s40779-016-0091-4)
Supplement: Additional file 2: — The Rivermead Post-concussion Symptom Questionnaire (RPSQ). (DOC 1078 kb) [file 40779_2016_91_MOESM2_ESM.doc]

Appendix 2. The Rivermead Post-concussion Symptom Questionnaire (RPSQ).


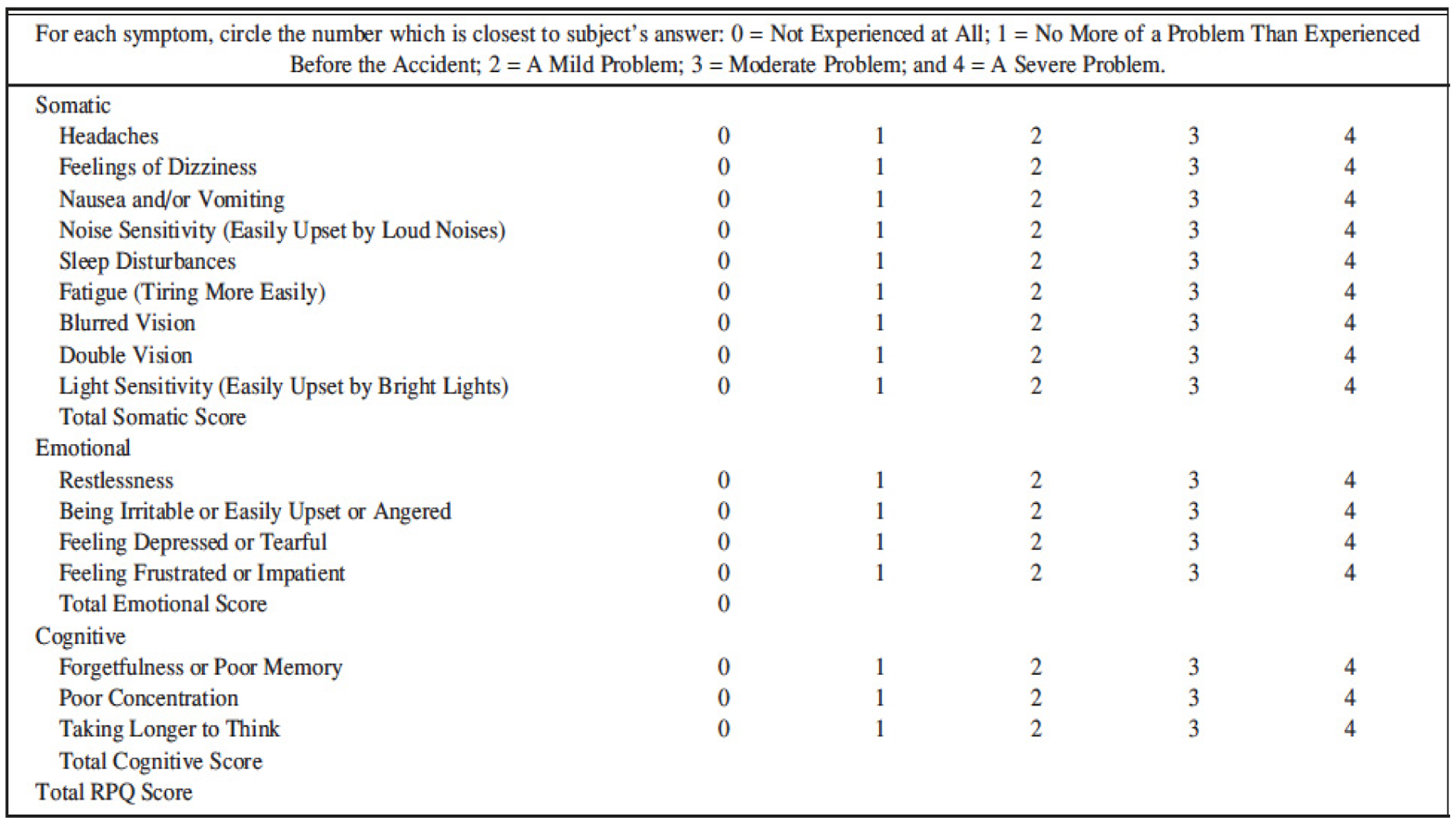


Reproduced from Springer Science + Business Media: King NS, et al: The Rivermead Post Concussion Symptoms Questionnaire: a measure of symptoms commonly experienced after head injury and its reliability. J Neurol 1995; 242(9): 587-92.
