# Supplementary figures and images for: The value of neurocognitive testing for acute outcomes after mild traumatic brain injury
Source: Mil Med Res. 2016 Jul 22;3:23. doi: 10.1186/s40779-016-0091-4 (PMC4957408; doi:10.1186/s40779-016-0091-4)

**Appendix 3.** The Mini Mental Status Examination (MMSE).


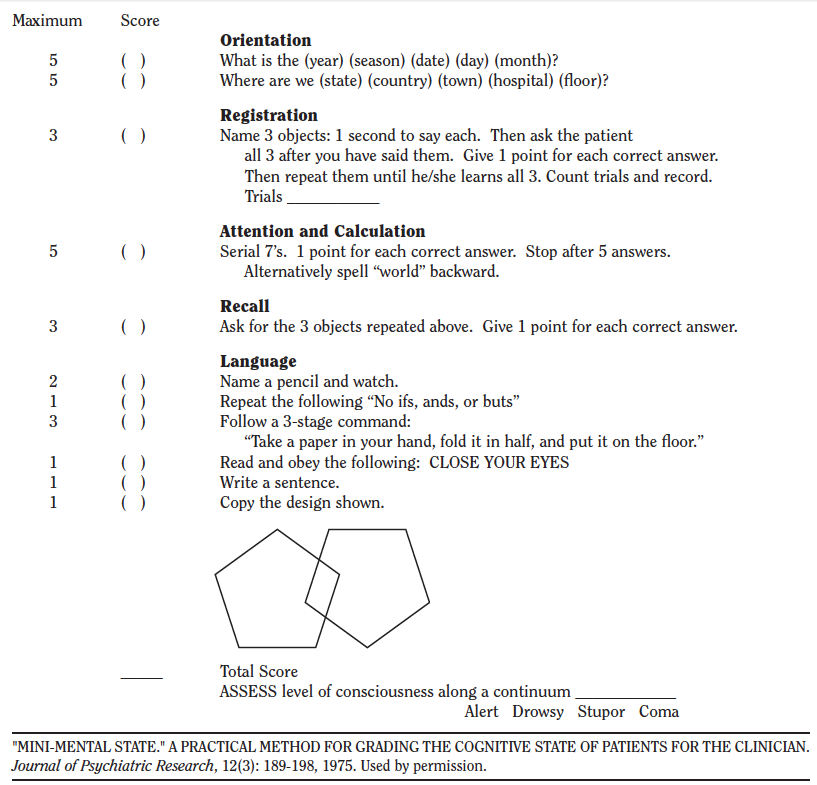

Supplement: Additional file 3: — The Mini Mental Status Examination (MMSE). (DOC 246 kb) [file 40779_2016_91_MOESM3_ESM.doc]

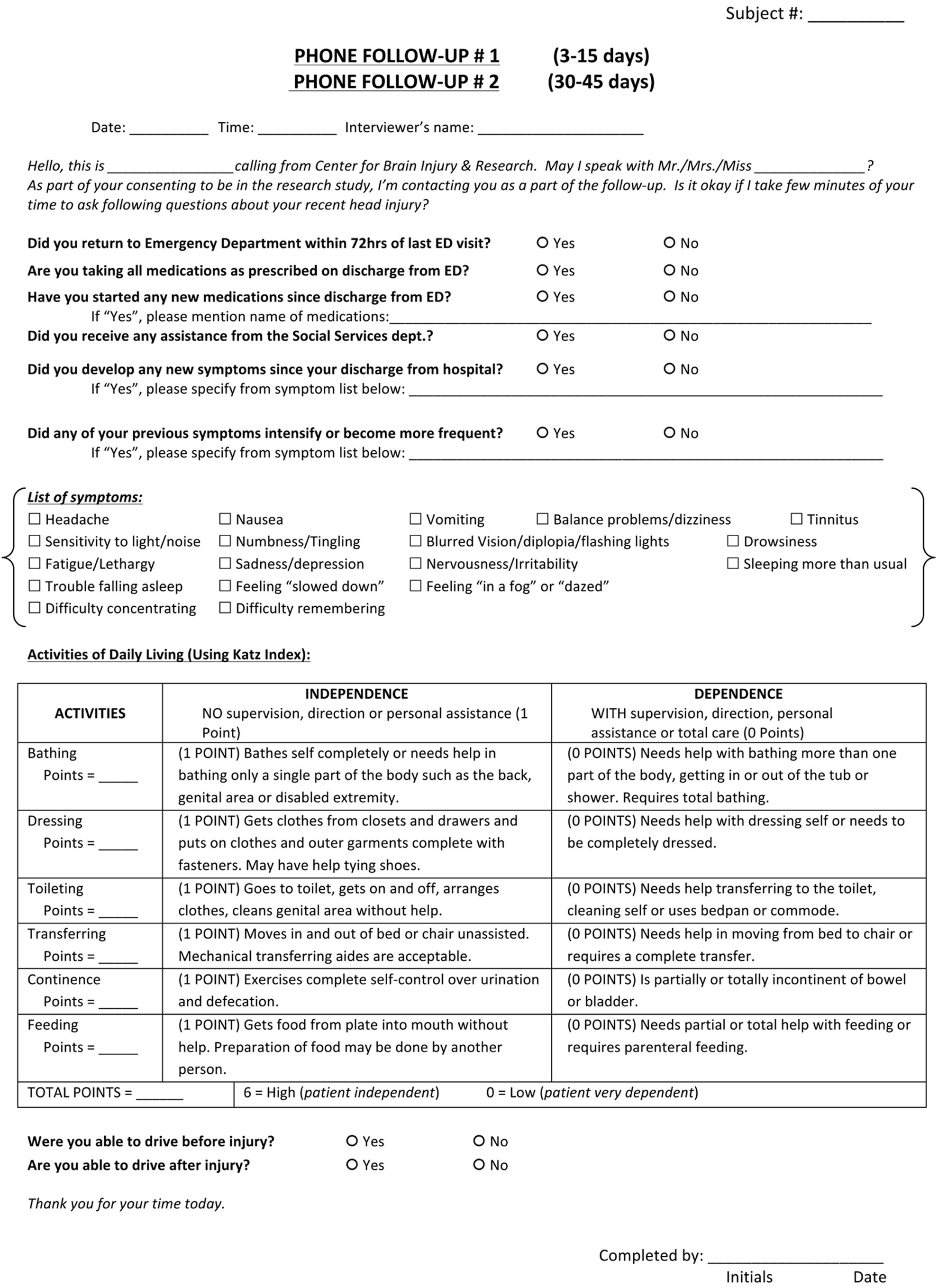

Supplement: Additional file 4: — Phone script for telephone follow-up. (TIFF 1798 kb) [file 40779_2016_91_MOESM4_ESM.tiff]
